# An Introduction to Practical Chemistry, Including Analysis

**Published:** 1857-02

**Authors:** 


*An Introduction to Practical Chemistry, including analysis.* By John E. Bowman, F. C. S. Second American, from the second London Edition. Philadelphia: Blanchard & Lea. 1856.

A valuable treatise for the working chemist, or for the medical man engaged in the chemistry of disease.

For sale by Phinney & Co.

